# Strength Properties of Structural Glulam Manufactured from Pine (*Pinus sylvestris* L.) Side Boards

**DOI:** 10.3390/ma14237312

**Published:** 2021-11-29

**Authors:** Radosław Mirski, Dorota Dziurka, Marcin Kuliński, Adrian Trociński, Jakub Kawalerczyk, Ryszard Antonowicz

**Affiliations:** 1 Department of Mechanical Wood Technology, Poznań University of Life Sciences, ul. Wojska Polskiego 28, 60-627 Poznań, Poland; radoslaw.mirski@up.poznan.pl (R.M.); marcin.kulinski@up.poznan.pl (M.K.); adrian.trocinski@up.poznan.pl (A.T.); jakub.kawalerczyk@up.poznan.pl (J.K.); 2 Faculty of Civil Engineering, Wrocław University of Science and Technology, Wybrzeże Wyspiańskiego 27, 50-370 Wrocław, Poland; ryszard.antonowicz@pwr.edu.pl

**Keywords:** structural glulam elements, structural beams, side boards, strength properties

## Abstract

The aim of this study was to assess the static bending strength of pine glulam manufactured when obtaining the main yield, i.e., structural timber or timber to be used in the production of structural glulam. Analyses were conducted on pine timber harvested from three different locations in Poland. Two beam variants were manufactured, differing in the timber arrangement, horizontal vs. vertical. It was shown that the static bending strength of beams manufactured in the vertical timber arrangement variant is slightly higher than that of beams produced from horizontally arranged layers, with the latter beams characterised by a smaller confidence interval for this strength. Moreover, it was found that the difference in the value of the 5th percentile for both beam types is slight and both beam types are considered to exhibit a high bending strength of over 40 N/mm^2^.

## 1. Introduction

Literature concerning timber obtained from tree stems, timber conversion methods and analyses of structural elements manufactured from timber is vast, while the wide range of materials obtained from various tree species is being expanded rapidly and it is invariably considered very attractive. This is closely related to the marked interest in such a modification of construction structures so that they meet the principles of the sustainable construction industry. For this reason, all initiatives and actions aiming at the utilisation of natural, truly eco-friendly materials which may be further re-used are considered to be so valuable. Wood is definitely a material fully meeting these assumptions and thus it is increasingly popular in building construction [1,2,3]. Steele [4] at the stage of conversion of logs into sawn timber indicated several groups of parameters affecting the yield of sawn timber from logs. These parameters include log diameter, length, tapering and quality; saw kerf width, conversion method, the share of wet and dry wood, decisions made by the sawmill workers, the condition of the saw itself and used equipment. Baltrušaitis and Pranckevičienė [5] analysed the profitability and yield of various log conversion methods, particularly for logs of small diameters. They indicated errors in the positioning of the log during conversion operations as the main parameter reducing yield. Rongrong et al. [6] proposed a new conversion method for small diameter logs and bonding of timber into structural elements. At present machine stress grading as well as advanced tools and statistical models are increasingly common and concern assessment of structural timber at each stage of its harvesting, starting from the appraisal of suitability of growing timber, through grading and appraisal of the raw material, i.e., logs and sawn timber, and ending with the manufactured products, such as, e.g., glulam beams. Grading principles for round wood and sawn timber, both for visual and machine grading, are defined in respective national standards, while in the case of the EU member countries these principles are specified in the EU standards supplemented with national appendices. Machine grading of timber typically consists of the determination of the modulus of elasticity (MOE), modulus of rupture (MOR) and density of a given element, with standing timber being evaluated visually or using non-destructive methods. In the EU countries many tree and wood species, both hardwood and softwood, have been tested and graded, while their suitability for the manufacture of solid structural glulam has also been assessed. This has been done, e.g., for Douglas fir originating from Ireland and the United Kingdom [7,8] and Germany [9], pine [10,11,12,13,14] and other softwood species [15,16] growing in Poland [10,17], while in the case of non-European countries it was, e.g., ponderosa pine from the USA [18], maple [19], black spruce from Canada [20], acacia from Indonesia [21] and Japanese larch [22,23]. Glulam beams may be manufactured from lamellas bonded horizontally or vertically. A vast majority of engineering applications are based on bent elements bonded in the horizontal lamella arrangement system. This results from a more advantageous distribution of stresses in the cross-section of a bent element under tensile stress [24]. Martins et al. [25] estimated strength parameters of sawn timber and bonded beams manufactured from Australian blackwood growing in Portugal and compared them with those of other hardwood species. Solid beams and bonded beams with varying numbers of lamellas manufactured from rubber tree wood were tested by Nadir and Nagarajan [26], who found no marked differences in strength and rigidity of solid vs. bonded beams and additionally described failure mechanisms. In turn, Mirski et al. [12,13] investigated the quality of sawn timber from pine trees growing in various geographical regions of Poland and obtained from logs cut from different stem sections. The applied conversion method was consistent with the practice adopted in Poland for structural timber. Those researchers also tested bonded beams manufactured from pine sawn timber [14]. In one of their studies [12], they presented, e.g., strength classification of sawn timber, while another paper [13] compared sawn timber strength grades according to national and EU standards. Moreover, causes for considerable discrepancies between them were analysed. In the opinion of the authors of this study, a valuable aspect was connected with the presented analysis of beams with the vertical lamella arrangement. While a large number of publications concern bent beams, relatively few studies investigate bending of elements with sawn timber arranged vertically at the cross-section (i.e., arrangement parallel to the plane of loading). Literature on the subject concerning such elements from bonded wood is scarce. A certain analogy to such structures may be found in bent elements manufactured from wood-based materials with the vertical arrangement of layers, such as laminated veneer lumber (LVL), with or without reinforcement [27,28,29], elements manufactured from cross laminated timber (CLT) [30,31], which in various structures are applied both flat and edgewise, as well as all types of I-beams, in which the web is found in the plane of loading [32]. Numerous studies present research results for such structures. Flaig and Blaß [30] analysed and tested beams from cross laminated timber (CLT) manufactured from spruce with lamellas in the cross arrangement system, in which thicker lamellas in the vertical system were bonded lengthwise using finger joints. Prior to bonding the density and the dynamic modulus of elasticity of lamellas were determined and the presence of wood defects (knots) was checked, which facilitated a visual classification of wood grades in the lamellas. Both beams and lamellas themselves were subjected to 4-point bending. The results confirmed, e.g., an advantageous effect of homogeneity of bonded beams and their increased mean bending strength compared to that of lamellas. Analogies to bent beams loaded parallel to the arrangement of lamellas or planks may also be observed in wooden (CLT) structures [33] used in the construction of small bridges. Structural elements with vertical lamellas at the cross-sections bonded mechanically were investigated in several studies [34,35,36,37]. However, the scope of the above-mentioned studies does not fully reflect the behaviour of bent beams with vertically bonded lamellas.

Thus it seems that the discussed problem needs to be analysed in more detail, since the thickness of side boards is much smaller than that of the main yield. For this reason, its use in beams in the horizontal system results in a considerable increase in the number of glue lines, which leads to greater amounts of adhesive used at the simultaneous lower yield of obtained sawn timber. The aim of this study was to assess static bending strength in bonded elements manufactured from pine sawn timber originating from the side yield used in the production of bonded structural elements or structural sawn timber.

## 2. Experimental Material

**Figure 1:**
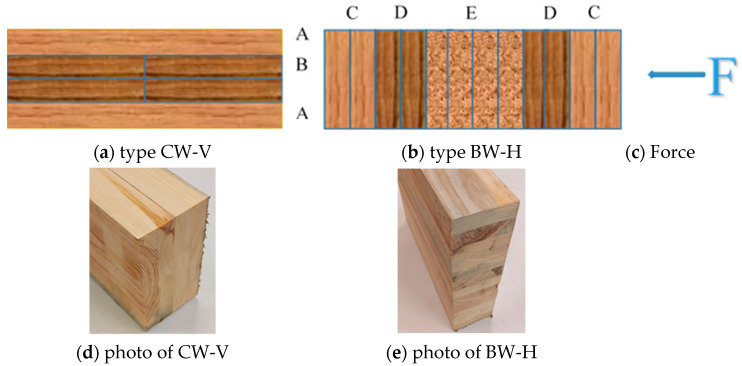
Sawn timber arrangement in the manufacture of structural beams (A,B,D,E), C—direction of loading; ((**a**)—quality in the bend test, (**b**)—waste timber, unclassified, (**c**)—visual assessment-class KW, (**d**)—visual assessment-class KS, (**e**)—visual assessment-class KG).

Analyses were conducted on pine sawn timber of 170 mm and 85 mm (width) × 19–25 mm (thickness) × 3485 mm (length). Sawn timber originated from the conversion of round wood obtained from three forest districts: Olesno (50°52′30″ N 18°25′00″ E), Wymiarki and Kalisz Pomorski. From those stands, lumber of 14.1 m in length were harvested, with round wood grades comparable to the average values of pine wood harvested at the rotation age in Poland. Grade WC0 accounted for 61%, WB0 for 32%, and WA0 accounted for 7%, respectively. The mean age of harvested trees was 109 years. Four logs of 3.5 m in length each were obtained from each analysed long log, with the sections marked as butt logs (B), middle logs (M)–2 pieces, and top logs (T). However, log marking was important only in the analyses of the main yield. From each log a cant was obtained, from which the main yield was derived to produce structural beams. Side boards of varying thickness and the opening plane width were produced both from the cant and the main yield. Next, side boards were edged, yielding edged timber of 170 mm, 140 mm, 120 mm, 100 mm, 85 mm and 70 mm in width and from 19 mm to 25 mm in thickness. Measurements showed that sawn timber of 170 mm and 85 mm in width has comparable thickness, thus it was decided to produce beams from this timber. Two variants with 8 sawn timber pieces per beam were obtained. It was the CW-V variant, in which the beam was composed of two pieces of wide sawn timber (170 mm) and 4 pieces of narrow sawn timber (85 mm) (Figure 1a) and BW-H, in which the beam consisted of 12 pieces of sawn timber of 85 mm in width (Figure 1b).

**Figure 2:**
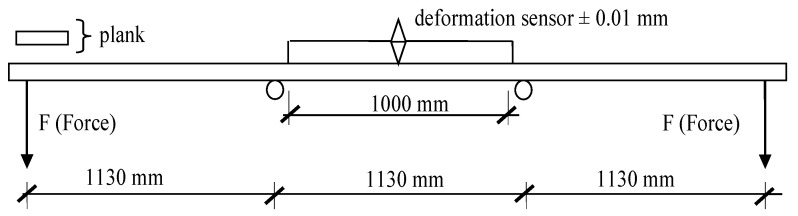
A diagram for assessment of the modulus of elasticity for investigated timber.

For each piece of wide sawn timber (over 100 mm), their linear dimensions, density and modulus of elasticity were determined. The modulus of elasticity was established based on the determination of the deflection under the assumed load according to the diagram presented in Figure 2. The sawn timber was tested flat, as in previous studies [13]. The initial load was assumed at 25.5 N (2.5 kg), at which the deformation sensor was set to zero and next load was increased to 147.15 N (15 kg). Timber was deflected 8 times, while deflection was recorded only for the last 5 measurements.

For the purpose of analyses from such graded timber, only the elements with the modulus of elasticity (E) ranging from 15 kN/mm^2^ to 19.7 kN/mm^2^ were used. The pieces were selected so that the faces were characterised by a comparable modulus of elasticity (E +/− 0.2 kN/mm^2^). Narrow timber (85 mm) was not tested and it was collected from the stack at random. It was assumed that narrow timber will have the modulus of elasticity ranging from 4.7 kN/mm^2^ to 7 kN/mm^2^, i.e., from the value of the 5% quantile to the mean value specified in the EN 338 standard [37] for timber grade C14. The equivalent value of the modulus of elasticity for the beam was calculated from Equation (1):

(1) $$E=V_{1}\cdot E_{1}+V_{2}\cdot E_{2}$$

where:

*V*—percentage share of a given phase,

*E*—Young’s modulus (modulus of compression-tension of a given phase.

Thus the modulus of elasticity for such manufactured beams should range from 9.85 kN/mm^2^ to 13.45 kN/mm^2^ and under advantageous conditions such beams should meet the requirements for glulam grade GL24c elements, since the mean value for this range of values is 11.65 kN/mm^2^.

In the second variant, timber was graded only based on visual inspection according to the PN-EN-518:2000/PN-D-94021:2013–10 standard [38,39]. However, assessment was conducted only for the distribution of such defects as knots, cracks, resin pockets, rot, wanes, cross grain and curvatures. Elements containing rot, considerable curvature (over 2 cm/m) as well as large areas of resinosis were not classified to any timber grade. The other pieces were classified to one of the three grades, i.e., KW (superior grade), KS (medium grade) or KG (inferior grade). Timber elements were classified to these grades corresponding to strength grades so that grade KW is ascribed grade C30, KS C24, while grade KG is equivalent to strength grade C18. Beams were manufactured in the symmetrical system, i.e., with 4 pieces from each grade. Assuming that KW timber is characterised by the modulus of elasticity of 12 kN/mm^2^, KS 11 kN/mm^2^ and KG 9 kN/mm^2^ (EN 338 [37]) and applying the known Equation (2) [14]:

(2) $$E_{ef}=\frac{1}{J_{x}}\overset{k}{\underset{i=1}{\sum }}E_{i}\left[J_{xi}+A_{i}{\left(d_{i}\right)}^{2}\right]$$

where:

*E_ef_*—effective/substitute modulus of elasticity, N/mm^2^,

*J_x_*—area moment of inertia, mm^4^,

*E_i_*—modulus of elasticity of layer, N/mm^2^,

*A_i_*—cross-sectional area, mm^2^,

*d*—distance from the neutral axis, mm.

The expected modulus of elasticity should be 11.63 kN/mm^2^. Thus both variants should exhibit similar ranges of strength and modulus of elasticity.

**Figure 3:**
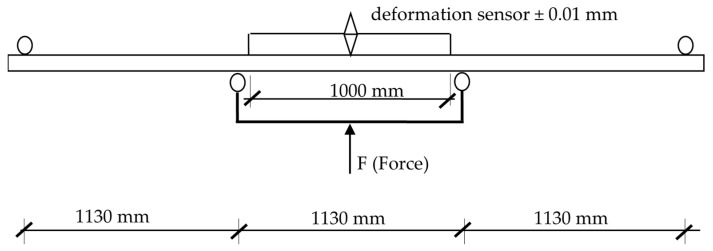
The diagram for loading of bonded beams.

Prepared sets directly before being bonded to form beams were dressed in order to ensure better quality of the bonded surface. The effective thickness of individual lamellas was 22.4 mm. Adhesive was applied on such prepared surface at 220–250 g/m^2^. Melamine-urea-formaldehyde resin MUF 1247 was used as a bonding agent together with the dedicated hardener 2526. Both products were manufactured by Akzo Nobel (Amsterdam, Netherlands). The mixture was prepared taking into consideration the conditions found in the laboratory room. It was assumed that 20 g of the hardener need to be added to 100 g resin. The adopted amount of the hardener is consistent with the recommendation for this resin by Akzo Nobel. The adhesive was applied using a glue roller applicator. Manufactured beams were subjected to a 4-point bending strength test according to the diagram presented in Figure 3. Beams were tested in the upright position.

During the failure test the site and cause of failure of a given beam were also investigated. Recorded results of direct measurements were subjected to statistical analysis using the STATISTICA 13.0 package (Version 13.0, StatSoft Inc., Tulsa, OK, USA). A total of 40 beams were manufactured, i.e., 24 CW-V type and 16 BW-H type beams.

## 3. Results and Discussion

**Figure 4:**
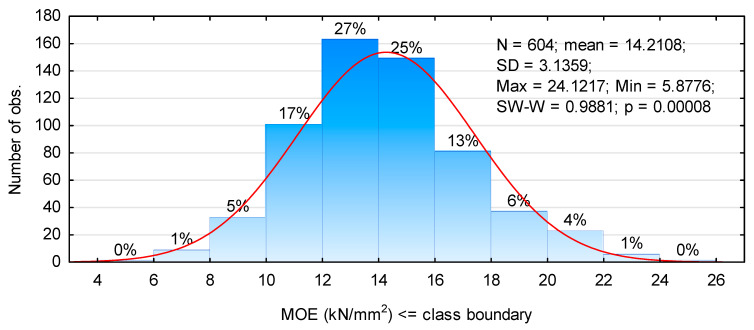
Histogram for the distribution of the modulus of elasticity.

Timber used in this study, in most sawmills, is treated as general purpose material. It is assumed that it is sawn timber of lower quality mainly due to the log section, from which it is collected. It is typically obtained in the range of thickness below 25 mm (after drying). This is the material collected the closest to the circumference of round wood and, for this reason, only smaller opening face widths may be obtained and usually, they vary in width over their length. Thus edging of this timber, particularly when it is to be of considerable length, results in significant material losses. Moreover, depending on the log position over the length of the long log the quality of this timber varies greatly, since often in the butt end the number of knots is lower and they are more often rotten, whereas the top section contains a large number of knots, but they are healthy. It results from the analysis of side boards conducted for the purpose of this project that the modulus of elasticity (in the tested batch) ranged from 5.7 kN/mm^2^ to 24.1 kN/mm^2^ (Figure 4). It should be noted that the analysis was done only for wide boards. However, it does not have a normal distribution, although the median is 13.96 kN/mm^2^ and it is only slightly lower than the mean (14.21 kN/mm^2^). Skewness is 0.405 and kurtosis is 0.202. Over 50% of the batch is timber with the modulus of elasticity between 12 kN/mm^2^ and 16 kN/mm^2^ and these are very high values, while only approximately 1% tested pieces had the modulus of elasticity below 8 kN/mm^2^ in the case of side boards with width exceeding 100 mm.

**Figure 5:**
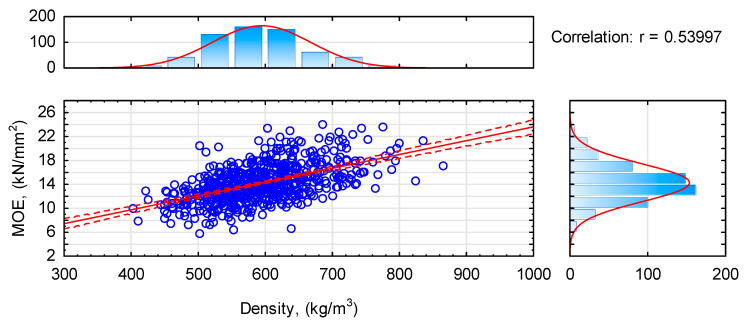
The effect of density on the modulus of elasticity determined in a 4-point bending test.

**Figure 6:**
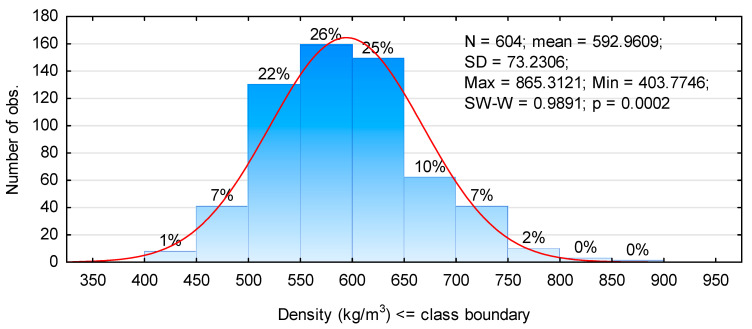
Histogram of the distribution of density.

High values of the modulus of elasticity are probably connected with the high density of the tested timber pieces. Although the effect of density on the modulus of elasticity is not as marked as it had been expected, since r is only 0.53997 (Figure 5), the trend is clear. Presented values of density represent density determined at 12% humidity, i.e., that corresponding to 20 °C and 65% RH. The moisture content of the tested timber pieces was characterised by mean moisture content close to that assumed, as it amounted to 11.4%. Thus in relation to the PN-EN 338 standard [34], the analysed timber batch meets the requirements for timber grade C50, since the mean density is 590 kg/m^3^ and the characteristic value (5-percentile) is 480 kg/m^3^. Moreover, almost 75% of tested timber pieces had a density between 500 kg/m^3^ and 650 kg/m^3^, while only 8% of pieces had a density below 500 kg/m^3^ (Figure 6). However, according to the data given in Figure 5, timber with a density below 500 kg/m^3^ is characterised by the modulus of elasticity from 7 kN/mm^2^ to almost 15 kN/mm^2^ and these are relatively high values.

**Figure 7:**
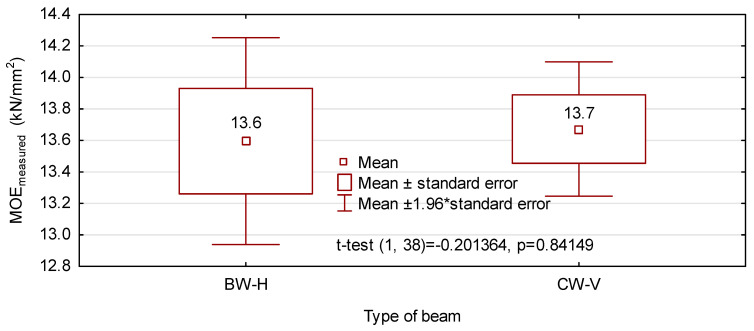
The modulus of elasticity of beams determined in the bending test.

**Figure 8:**
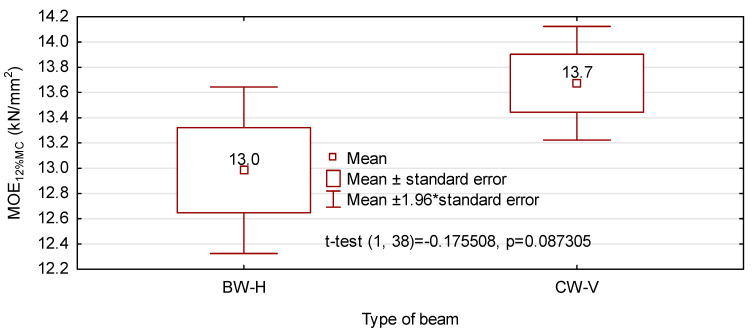
The modulus of elasticity of beams considering moisture content of beams during the test.

It results from the values presented in Figure 7 that both beam types exhibit an almost identical value of the mean modulus of elasticity, which is consistent with the preliminary assumption. According to the conducted statistical analysis, the 95% confidence interval for CW-V beams is much smaller than that for BW-H beams. Thus the results closer to the mean are obtained for beams manufactured from 8 timber layers and analysed in the vertical system. When the moisture content of tested beams is considered, i.e., it is converted to 12% MC (using Bauschinger’s Equation (I) [13]), BW-H beams are characterised by a slightly lower modulus of elasticity (Figure 8). Based on the data given in Figure 8 there are no grounds for the rejection of the zero hypothesis talking about the equality of mean moduli of elasticity for both beam variants. What is important, values of the modulus of elasticity obtained in this analysis are by approx. 15% higher than those initially assumed. In the case of CW-V beams, this may result from a higher quality of pine sawn timber than it had been originally assumed.

It results from data presented earlier that the mean modulus of elasticity for side boards is approximately 14 kN/mm^2^ (Figure 4), thus it is 2-fold higher than it had been assumed. However, when assuming the mean value the modulus of elasticity when calculated according to formula 1 and beams of type CW-V at approximately 15.7 kN/mm^2^ should be expected, whereas the obtained value is much lower. Nevertheless, the modulus of elasticity was assessed only for sawn timber with width over 100 mm. Wider sawn timber probably originates from deeper located log layers, thus it may exhibit better mechanical properties. In the latter case, i.e., BW-H beams, the assumed values of the modulus of elasticity for a given grade during visual grading are underestimated. However, it may be assumed that this difference, amounting, for these beams, to approximately 10%, is a factor increasing certainty of the required quality for the manufactured elements. In the case of glulam elements and the modulus of elasticity evaluated according to the PN-EN 14080:2013 standard [40], not only the mean modulus of elasticity is assessed, but it is also the value of the 5th percentile. In the case of the analysed beams, these values are 11.3 kN/mm^2^ and 12.1 kN/mm^2^ for BW-H and CW-V beams, respectively.

**Figure 9:**
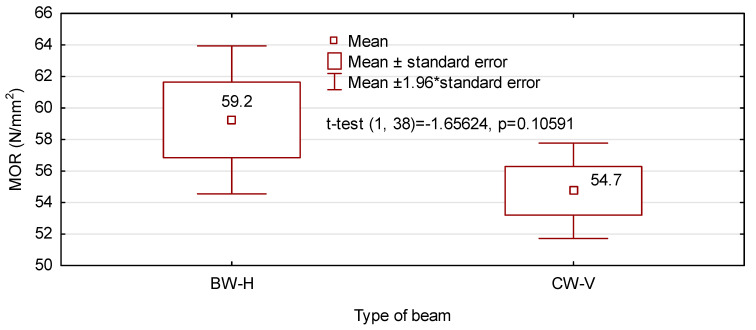
Static bending strength.

Slightly higher values of the modulus of elasticity for CW-V beams are not reflected in greater static bending strength. The mean strength of CW-V beams is 54.7 N/mm^2^, while for BW-H beams it is 59.2 N/mm^2^ (Figure 9). Although the difference is approximately 8%, there are no grounds to state that both batches differ statistically. Recorded values are very high because they account for approximately 50% bending strength of pine wood. However, a statistically significant value for bending strength is the value of the (5-percentile). In the case of analysed beam types, these values amount to 42.0 N/mm^2^ and 43.2 N/mm^2^ for BW-H and CW-V beams, respectively. In both cases characteristic bending strength is also very similar; nevertheless, a more advantageous value for obtained for CW-V beams. Thanks to such ranges of strength and the modulus of elasticity both beam types are characterised by a greater load carrying capacity than that required for GL24c beams.

Considering only these two parameters BW-H beams may be classified as grade GL30c, while CW-V beams as grade GL32c. It turns out that irrespective of the type of the distribution measure to be analysed, the recorded bending strength values are much higher than those obtained in tests conducted on the main yield presented in a study by Mirski et al. [14], whereas they are comparable to those for rod-reinforced beams [41].

**Figure 10:**
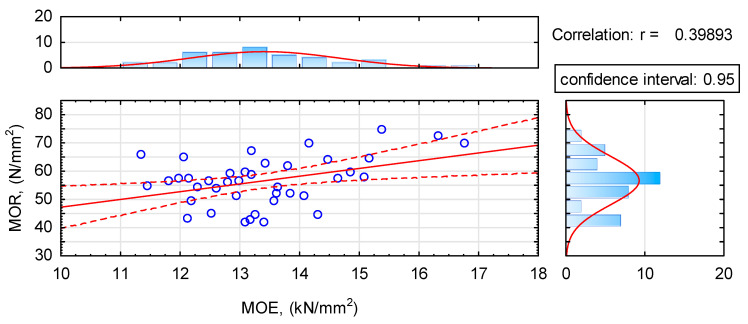
The dependence of strength on the modulus of elasticity.

What is significant, in the case of analysed beams, the modulus of elasticity is not a definite descriptor of static bending strength. Although a trend towards an increase in strength with an increase in the modulus of elasticity, the value of the linear correlation coefficient is only 0.4 (Figure 10). Thus it seems that a decisive role is played by the distribution of defects, which had not been removed in accordance with the concept of this project.

## 4. Conclusions

Pine timber is readily available in Europe; however, it is not as popular in wooden building structures, particularly in glulam production, as is the case with spruce timber. Although mechanical properties of pine wood are considered to exceed those of spruce wood, due to the appearance of knots and their distribution on the side of the structural material this timber is not as highly valued as spruce timber. When preparing pine sawn timber either in the form of solid structural elements or those intended for bonding into glulam the so-called side boards are produced. The amount of side boards depends on many factors; nevertheless, it may be assumed that it is approximately 20%. Frequently it is sawn timber with extremely differing quality properties; however, it results from the conducted analyses that a valuable structural material may be manufactured from side boards. Specific conclusions of this study include as follows:

- the modulus of elasticity of side boards falls within a very wide range of values, from approx. 5.5 kN/mm^2^ to 24 kN/mm^2^; however, over 60% of the material is sawn timber with the modulus of elasticity over 11 kN/mm^2^,
- side boards are characterised by high elastic properties,
- in accordance with the assumptions, both variants were characterised by a comparable modulus of elasticity, although it was much higher than it had been expected,
- static bending strength of beams manufactured in the vertical timber arrangement system is slightly higher than that of beams produced from horizontally arranged timber,
- beams manufactured from horizontally arranged timber layers shows a smaller confidence interval for static bending strength,
- the difference in the value of the (5-percentile) for both beam types is slight and it needs to be stated that both beam types exhibit a high bending strength exceeding 40 N/mm^2^.

## Data Availability

The data presented in this study are available on request from the corresponding author.
